# Health-care utilization of patients with chronic back pain before and after rehabilitation

**DOI:** 10.1186/s12913-017-2757-3

**Published:** 2017-12-06

**Authors:** Magdalena Görge, Jeanette Ziehm, Erik Farin

**Affiliations:** Section of Health Care Research and Rehabilitation Research, Medical Centre – University of Freiburg, Faculty of Medicine, University of Freiburg, Freiburg, Germany

**Keywords:** Chronic back pain, Rehabilitation, Health-care utilization, Sick leave

## Abstract

**Background:**

Patients with chronic back pain show an increased use of health-care services leading to high direct costs. Multidisciplinary rehabilitation reduces pain intensity, depression, disability and work inability. The study aims to investigate whether health-care utilization in patients with chronic back pain is lower after rehabilitation than before rehabilitation and if, in addition to sociodemographic, medical and psychological characteristics, changes in these characteristics immediately after rehabilitation can predict health-care utilization.

**Methods:**

*N* = 688 patients with chronic back pain were asked about their overall health-care services use and the use of general practitioners, specialists, physiotherapy, psychotherapy, complementary therapist, massages, and admission to hospital both 6 months before and 6 months after rehabilitation. In addition, various sociodemographic, medical and psychological variables were assessed. To measure changes due to rehabilitation, differences in pain intensity, disability, impairment and coping, quality of life, and days on sick leave before and after rehabilitation were calculated. Dependent t-tests and hierarchical regression analyses were used to analyse the data.

**Results:**

Health-care utilization 6 months after rehabilitation was, except for physiotherapy and psychotherapy, significantly lower than before. The effect sizes were rather small (Cohens’*d* =. 01–.34). After rehabilitation between 15.2% and 39.9% of the variance of health-care utilization could be explained. The baseline values of health-care utilization explained between 3.2% and 15.9% of the incremental variances. The changes in psychological impairment and coping as well as in sick leave after rehabilitation could explain between 0.8% and 2.9% of the variance of health-care utilization after rehabilitation. Its influence was significant for the general use of health-care services, general practitioners and specialists.

**Conclusions:**

The results indicate that use of health-care services after rehabilitation in the present study is slightly lower than before, which has an impact on direct costs due to chronic back pain in Germany. The predictors show the importance in terms of health-care utilization of improving work ability and psychological impairment.

## Background

Back pain is one of the most common health problems in Western societies. In Germany, lifetime prevalence for acute back pain is reported to be between 74% and 80% [1]. If duration of back pain exceeds 3 months, it is classified as chronic with a reported lifetime prevalence of between 10% and 27% [2]. Back pain affects health and quality of life but also influences functioning in everyday life, work and leisure. This often leads to inability to work and frequent use of health-care services resulting in high costs for insurances and society [1, 3].

Patients with chronic back pain use more health-care services than the average population [4]. Health-care utilization represents a complex pattern of individual and contextual characteristics, which are described in the widely used Behavioral Model of Health Services Use [5]. In this model, the characteristics are determined by predisposing, enabling and need factors. The Behavioral Model of Health Services Use [5] was the theoretical background for a representative survey about health-care utilization in Germany over a 12-month period [6]. The survey indicated that 97% of the population had at least one consultation with physicians per year. On average, people in Germany required 9.2 outpatient consultations within 12 months, with 3.2 of these being with a general practitioner. In addition, they had an average of 2.6 meetings with physiotherapists per year, visited by around a quarter of the population [6]. Patients with acute and chronic back pain consulted a general practitioner, on average, 4.8 times over 6 months. Forty-one per cent of these patients also attended a specialist over the same 6 months [7].

State of health has been identified as one of the most important predictors of outpatient medical health service use [6]. People with poor health usually had more health-care utilization. Only preventive medical services like dentists were consulted more often by persons with a good state of health. Other important predictors for health-care utilization were gender, age, social status and health insurance [6]. Women used health-care services more often than men, but the gender difference decreased with age [6, 8]. With regard to patients suffering from low back pain, women showed a higher health-care utilization than men, but chronification, disability, and depression were more important than gender differences to predict the use of health-care services [4, 9].

Rattay and colleagues showed that people required more in- and outpatient health-care services with age [6]. People older than 50 with a lower socio-economic state reported less consultations with specialists than patients with a higher socio-economic state, of the same age and state of health. In contrast, they were more likely to contact a general practitioner [10]. Elderly patients with chronic back pain, a high pain intensity and co-morbid depression had the highest rate of consulting physicians and physiotherapists, the highest rate of hospitalization, and utilization of medication compared to other patients with back pain [11]. Additionally, for patients suffering from back pain, psychosocial factors such as anxiety, depression, fear-avoidance beliefs and pain-related stress seem to be important predictors for health-related quality of life and health services utilization over a period of 6 months [12].

A systematic review [13] revealed that the effects of a multidisciplinary rehabilitation on health-care utilization in patients with chronic back pain have rarely been investigated, although they have a large impact on society and costs. Multidisciplinary rehabilitation seems to have small to moderate effects on pain intensity, disability and the ability to work. As a consequence of the regulation and financing of rehabilitation, the general duration of rehabilitation treatment in Germany is 3 weeks. Inpatient and outpatient rehabilitations for patients with chronic back pain focus on reduction of pain and disability, functional coping, psychosocial stress reduction, and prevention of impairment in social and working life. The rehabilitation is conducted by a multidisciplinary team including physicians, psychologists, physiotherapists and occupational therapists [14]. The multimodal programme contains somatic, educational, psychotherapeutic, social and occupational therapy, such as physical training, relaxation and coping strategies. A review indicated moderate short-term evidence for rehabilitation in patients with chronic back pain in Germany regarding pain intensity, vitality, depression, disability and coping [15]. There is also moderate to strong evidence that intensified behavioural health rehabilitation influences pain intensity and state of health, especially when a psychological intervention is included [16]. The effects of rehabilitation in Germany on inability to work have rarely been investigated and have been reported as small [15]. However, patients with back pain who stayed at work reported lower pain intensity, less pain duration, more pain acceptance, better mental health, lower psychological distress and a lower perceived workload than patients on sick leave [17].

Patients with chronic pain in Sweden were able to reduce health-care utilization from 27.4 consultations to 20.4 consultations during 12 months after multidisciplinary rehabilitation [18]. Other Scandinavian studies also showed decreased health-care utilization in patients with chronic back pain over 4, 12 and 24 months as well as reduced days on sick leave after a multidisciplinary rehabilitation or a physiotherapeutic intervention [19, 20].

Due to the lack of specific studies concerning the German health-care system, this study aims to investigate whether there is a difference in health-care utilization before and after a multidisciplinary rehabilitation in patients with chronic back pain in Germany and whether, in addition to sociodemographic, medical and psychological characteristics, changes due to rehabilitation determine health-care utilization after rehabilitation.

We presume that overall health-care utilization and the use of general practitioners, specialists, hospital, physiotherapy, psychotherapy, massages, and complementary therapists during the 6 months after rehabilitation in Germany will be significantly less than in the 6 months before rehabilitation [18–20]. The reduction in pain intensity, function and disability, and days on sick leave as well as improved health-related quality of life and coping with pain immediately after rehabilitation will significantly account for variance in health-care utilization. Furthermore, we hypothesize predisposing sociodemographic characteristics (gender, age, education, income, ability to work and sick leave during the 6 months after rehabilitation, etc. (e.g. [6])), medical characteristics (chronification, pain intensity, disability, co-morbidities [9, 11]), psychological variables such as coping with pain, and pain-related cognitions [12] as well as the self-evaluated state of health [6], measured immediately after rehabilitation, will explain health-care utilization in patients with chronic back pain. We expect lower age, being male [6], ability to work, low pain duration, low pain intensity [17], good state of health [6], functional coping with pain, low disability and functional pain-related cognition [15] to lead to a lower health-care utilization.

## Methods

### Design and setting

This is a secondary analysis of data surveyed within the framework of the project ‘Patient–provider communication for chronically ill patients: gender and age-specific preferences of patients’, which has been approved by the ethics committee of the University of Freiburg (approval number 62/08, (see [21])). Patients with chronic back pain in four inpatient and seven outpatient rehabilitation centres in different regions of Germany were surveyed. The mean length of rehabilitation in this sample was *M* = 20.62 days and was relatively homogeneous, *SD* = 4.5. More information about the rehabilitation programme are described in the background section.

The patients were asked to fill out questionnaires at the beginning of the rehabilitation, at the end of rehabilitation, and 6 months after rehabilitation. In addition, the responsible physician in the rehabilitation centre completed a documentation sheet with characteristics of the disease and motivation for each patient.

### Participants

Patients with chronic low back pain who were undergoing rehabilitation were surveyed. In Germany, both inpatient and outpatient rehabilitation centres provide rehabilitation for back pain patients and the therapy programmes of both types of institutions are very similar. Four inpatient and seven outpatient orthopaedic rehabilitation centres participated in this study. The goal of multidisciplinary pain treatment is to prevent or mitigate impairment of participation in working and social life. The German National Disease Management Guideline envisions multidisciplinary rehabilitation involving various professional groups (e.g. physicians, physiotherapists/sports therapists, psychologists, occupational therapists). The multimodal programmes include educational, somatic, psychotherapeutic, social and occupation-related therapy. Examples of treatment elements are information (e.g. providing information on chronic back pain and rehabilitation goals in educational group sessions), training based on a biopsychosocial disease model, occupational therapy, physical therapy, exercise therapy, and psycho-therapeutic treatment to modify maladaptive illness behaviour and learn techniques for relaxing and coping with stress. The treatment team is headed by a physician. The patient generally has 4–5 therapy sessions a day on workdays.

The criterion for inclusion was non-specific chronic back pain for at least 6 months. Patients were excluded if chronic back pain was due to inflammatory disease or tumour. The questionnaires were consecutively given to all patients who were able and willing to participate (informed consent). Altogether *N* = 1039 were asked to participate and *N* = 701 agreed. Due to data inconsistencies 13 participants had to be excluded, so that the final sample contained *N* = 688 participants. The percentage of decliners was 32.5%. The main reasons for non-inclusion were refusal to participate, cognitive or physical limitations, and language problems. For 52 patients no reason for non-inclusion was reported. The dropout rate after rehabilitation was 8.9%. Six months after rehabilitation the dropout rate was 30.9%. The patients who remained in the study were significantly older, less often employed, less often on sick leave, had a better state of health and fewer consultations with specialists. They were also less often admitted to hospital compared to the dropout patients. Information on the patients in the study is described in Table 1.

**Table 1:** Respondent characteristics (*N* = 688)

Age (*M; SD*)	51.0 (11.2)
Gender (% female)	57.2
Marital status (% married)	61.1
Level of education (%)	
Elementary school	31.5
Secondary school	50.8
University-entrance diploma	12.2
Employment (%)	72.3
Inability to work (%)	37.7
Income per month (%)	
< 500 euro	2.9
500–1000 euro	11.5
1000–1500 euro	21.4
1500–2000 euro	25.3
2000–2500 euro	12.5
2500–3000 euro	11.8
3000–3500 euro	6.1
> 3500 euro	8.6
Pain intensity (VAS; 0–100, *M; SD*)	52.9 (22.7)
Chronification (%)	
Acute event	0.6
< 1 year	12.4
1–2 years	11.1
3–5 years	18.6
6–10 years	16.3
> 10 years	40.2
Treatment motivation (1–6, *M; SD*)	5.2 (0.88)

### Instruments

Co-morbidities [22], treatment motivation and illness duration were assessed by the questionnaire for physicians. The questionnaires for patients included sociodemographic information (age, gender, marital status, education, employment, income, ability to work, sick leave, hours of work, state of health) as well as medical and psychological variables. The intensity of pain was quantified by the Visual Analogue Scale (VAS, range 0–100, 0 = no pain). Pain function and disability due to back pain were measured using the Oswestry Disability Index (ODI [23]; range 0–100, 0 = no disability). To record pain-related impairment and coping, the German pain-coping Questionnaire (FESV) [24] was used. The questionnaire measures three constructs each including three scales. The construct psychological impairment consists of pain-related helplessness and depression, pain-related anger (range 5–30, 5 = low impairment), and pain-related anxiety (range 4–24, 4 = low anxiety, Cronbach’s alpha .88 to .93). The other two constructs are behavioural coping (rest and relaxation, mental distraction, and countersteering activities, range 4–24, 4 = low coping, Cronbach’s alpha in the present study .80 to .82) and cognitive coping (experience of competencies, cognitive restructuring and action planning, range 4–24, 4 = low coping, Cronbach’s alpha .79 to .85). Health-related quality of life was measured with the scales physical component (SF-12-PC) and mental component (SF-12-MC, range 0–100, 0 = lowest quality of life) [25]. All these variables were assessed at each measurement.

To record changes after rehabilitation, we computed the differences between the values of the scales VAS, ODI [23], FESV [24], SF-12 [25] before rehabilitation and at the end of rehabilitation, and in the number of days on sick leave before rehabilitation and 6 months after rehabilitation.

In addition, the psychological variables fear-avoidance beliefs, control beliefs, and illness coherence were assessed. Fear-avoidance beliefs were measured using the Fear-Avoidance Belief Questionnaire (FABQ) [26], which has two scales: physical activity (range: 0–6, 0 = low avoidance; Cronbach’s alpha = .79) and beliefs about work (Cronbach’s alpha = .88). The construct illness coherence was assessed with the Illness Perception Questionnaire (IPQ-R; range 5–25, 5 = lowest coherence, Cronbach’s alpha = .81) [27, 28]. To measure control beliefs, the control beliefs concerning illness and health questionnaire (KKG) was used [29]. The questionnaire consists of three scales: internal locus of control (LOC-I; Cronbach’s alpha = .82), social external locus of control (LOC-SE; Cronbach’s alpha = .67), and fatalistic external locus of control (LOC-FE; range 7–42; 7 = highest control belief, Cronbach’s alpha = .78;) The variables were measured at the beginning of the rehabilitation. All questionnaires were used in the respective German version.

### Outcome variables

The utilization of general practitioners, specialists, hospital, physiotherapy, psychotherapy, complementary therapist, and massage during the last 6 months was measured at the beginning of the rehabilitation and 6 months after rehabilitation using the patient’s questionnaire. To assess the overall health-care utilization, the number of contacts with all types of health care was summed up by an index of health-care utilization.

### Statistical analyses

#### Multiple imputation

Due to regression analyses with a large number of predictors all having some missing values, a case-wise deletion would bring many disadvantages. Hence, multiple imputation was used to replace missing values [30]. For each measurement all participants with more than 50% missing values were eliminated from the analysis, so that 440 participants remained. Due to software restriction the data set was separated into four parts (before rehabilitation, after rehabilitation, 6 months after rehabilitation, physician) to conduct multiple imputation. Auxiliary variables were used to have correlation among the four parts. Five imputed data sets were created for each part using expectation-maximization and data augmentation procedure integrated in NORM 2.03. The plots showed rapidly converging series and low autocorrelations. All further analyses were conducted with all imputed data sets. The relevant parameters (standard errors, regression coefficients etc.) were combined according to Rubin’s rules [31] to receive one estimate of parameters.

#### Descriptive statistics and mean differences

All outcome variables were analysed descriptively (mean, range, frequencies). To test the mean differences of outcome variables between the first measurement and 6 months after rehabilitation, t-tests for dependent samples were conducted. To assess the relevance of the differences, effect sizes were calculated, which were defined as the mean differences divided by the pooled standard deviation before rehabilitation and 6 months after rehabilitation. Effect sizes of 0.20 are considered as small, around 0.50 as medium, and 0.80 as large [32].

#### Hierarchical regression analyses

Hierarchical regression analyses were performed for each outcome variable (generalists, specialists, hospital, physiotherapy, psychotherapy, index of health-care utilization) 6 months after rehabilitation. In a first step, the baseline values for respective health-care utilization at admission were included in regression analyses. In a second step, all sociodemographic, medical and psychological variables as well as the characteristics of pain intensity, function of pain and disability, health-related quality of life and coping immediately after rehabilitation were added. In a third step, the differences in sick leave during the 6 months before and the 6 months after rehabilitation, in pain intensity, function of pain and disability, health-related quality of life and coping before and immediately after rehabilitation were included. A stepwise method of variables inclusion was conducted for all five imputed data sets (P_IN_ = .05; P_OUT_ = .10). Predictors included in the model in two or more of the five imputed data sets were supposed to be relevant. With this restriction the model could be sparser and multicollinearity could be prevented. Regression analyses were performed again using a forced entry method of variables inclusion. Only potentially relevant predictors were included in the model, so that the results of the five imputed data sets were directly comparable. To assess multicollinearity, the variance inflation factor (VIF) was calculated for the final models. Values higher than 5 can be considered as an indication of multicollinearity [33]. SPSS 23 was used for analyses.

## Results

Table 2 presents the descriptive statistics, mean differences of dependent t-tests and effect sizes for the outcome variables. Most of the patients consulted at least one type of health-care service before and after rehabilitation. The means of the index of health-care utilization, and the use of general practitioners, specialists, admission to hospital, massages and complementary therapist after rehabilitation were significantly lower than before rehabilitation. The means of utilization of physiotherapy and psychotherapy were lower after rehabilitation than before, but the differences were not significant. The effect sizes ranged between Cohen’s *d* = .01 (utilization of complementary therapist) and Cohen’s *d* = .34 (specialists) indicating small effects.

**Table 2:** Descriptive statistics, mean differences and effect sizes of outcome variables (*N* = 440)

	Before rehabilitation			After rehabilitation			Mean differences	
	At least 1 consultation (%)	Range	M (SD)	At least 1 consultation (%)	Range	M (SD)	*T*	Effect size (Cohens’d)
Index health-care utilization	99.7	0–151	23.24 (16.75)	93.9	0–146	19.41 (19.27)	3.806***	.21
General practitioners	94.6	0–120	5.22 (6.59)	85.9	0–48	3.76 (4.68)	4.276***	.24
Specialists	93.0	0–48	5.32 (5.48)	84.4	0–35	3.53 (4.51)	4.264***	.34
Admission to hospital	30.2	0–9	0.41 (0.82)	18.6	0–15	0.22 (0.87)	2.758**	.22
Physiotherapy	67.0	0–90	8.29 (0.16)	49.2	0–60	7.89 (0.26)	0.293	.22
Massage	45.9	0–50	4.08 (9.56)	34.8	0–60	3.24 (11.01)	2.675**	.04
Psychotherapy	16.3	0–48	1.01 (6.14)	17.8	0–25	0.99 (6.23)	0.288	.14
Complementary therapist	17.3	0–20	0.94 (3.539)	15.2	0–15	0.65 (3.08)	2.144*	.01

*M* mean, *SD* standard deviation; *** *p* < .001, ** *p* < .01,* *p* < .05

Table 3 shows the results of the hierarchical regression analyses. There was no indication of critical multicollinearity as the values of the VIF were mostly below 2 and never higher than 3. The assumption of homoscedasticity was violated, which could lead to biased significance. However, the regression coefficients were supposed to be valid [34].

**Table 3:** Hierarchical regression analyses (*N* = 440)

	Index health-care utilization	General practitioners	Specialists	Admission to hospital	Physio-therapy	Psycho-therapy
*β*						
Baseline values of corresponding utilization variable	.288***	.288***	.141**	.077	.165**	.219***
***R*** ^***2***^ ***after 1st step***	.128***	.159***	.078***	.025**	.032***	.056***
*β*						
Gender	−.158***				−.173**	
Inability to work					.139**	
Employment						−.085
Hours of work	−.122**	−.090*			−.095	−.078
Days on sick leave	.332***	.231**	.378***	.309***	.149*	.283**
State of health		.121*	.182**			
SF-12 PC		−.082				
Chronification		.071		−.109*		
Co-morbidity				.082		
Disability (ODI)						−.134
Helplessness and depression (FESV)	.187***				.167**	.208**
Anger (FESV)		.180**				
Anxiety (FESV)		−.091				
Experience of competencies (FESV)					.088	
Activity beliefs (FABQ)	−.081*				−.071	
LOC-FE	.097*				.085	
**Change** ***R*** ^***2***^ ***after 2nd step***	.252***	.218***	.248***	.115**	.148**	.119***
*β*						
Difference sick leave	−.144*	−.112*	−.146*		−.154	
Difference helplessness and depression			−.104*			
Difference anger						.088
Difference anxiety		.085*	.118*			
Difference experience of competencies		.072				
Difference cognitive restructuring				.093*		
Difference pain function and disability			.075			
Difference pain intensity				**.**058		
**Change** ***R*** ^***2***^ ***after 3rd step***	.015**	.019**	.029**	.012	.010	.008
***R*** ^***2***^	**.394**	**.399**	**.327**	**.152**	**.191**	**.182**

Standardized regression coefficients *β* and *R* ^*2*^ with significance, *** *p* < .001, ** *p* .01 , * *p* < .05; significant parameters are printed in bold type (*p* < .05)

Between 15.2% and 39.9% of the variance of health-care utilization in patients with chronic back pain 6 months after rehabilitation could be explained by the predictor variables. The respective baseline values of health-care utilization during the 6 months before rehabilitation explained between 2.5% and 15.9% of the variance of health-care utilization after rehabilitation. Except for admission to hospital, all baseline values were significant predictors. The amount of the explained incremental variance by the sociodemographic, medical and psychological variables ranged from 11.9% to 25.2%. The most important predictor for all types of health-care utilization was days on sick leave. Patients requiring more days on sick leave had a higher health-care utilization. The index of health-care utilization was also influenced by gender, hours of work, pain-related helplessness and depression, activity-related fear-avoidance beliefs and fatalistic externality of control beliefs. Being male, fewer hours of working, a higher score in pain-related helplessness and depression, fewer activity beliefs and low fatalistic externality of control beliefs favoured more health-care utilization in general. Within this block of variables, in addition to days on sick leave, hours of work, state of health and pain-related anger explained variance on the use of general practitioners. Persons with fewer hours of work, a worse state of health and more pain-related anger consulted more frequently with general practitioners over the 6 months after rehabilitation. Besides days of sick leave, utilization of specialists was also influenced by state of health. People with poor state of health consulted a specialist more often. A shorter duration of illness led to a higher frequency in admission to hospital. In addition to sick leave, other important predictors for physiotherapy in this block were gender, inability to work and pain-related helplessness and depression. Men who were unable to work with more pain-related helplessness and depression made more use of physiotherapy. Besides sick leave, pain-related helplessness and depression was a significant predictor of utilization of psychotherapy insofar as higher pain-related helplessness and depression was related to a higher claim on psychotherapy.

After adjusting for baseline values, sociodemographic, medical and psychological variables, the differences in sick leave, pain intensity, function of pain and disability, health-related quality of life and coping after rehabilitation, which are presented in Table 4, explained additionally between 0.8% and 2.9% of the variance. The incremental explanation of variance was significant for the index of health-care utilization, the use of general practitioners and specialists. Patients having a higher reduction in days on sick leave required health-care services, general practitioners and specialists less frequently. Patients with a smaller decrease in helplessness and depression after rehabilitation had more consultations with specialists. A greater reduction in anger due to rehabilitation led to a higher utilization of general practitioners and specialists.

**Table 4:** Differences in pain intensity, function and disability, coping, health-related quality of life and sick leave before and after rehabilitation

	Differences in raw score	Cohens d
FESV helplessness and depression	1.58***	.37
FESV anxiety	1.19***	.32
FESV anger	1.03***	.21
FESV action planning	−0.97***	.24
FESV cognitive restructuring	−0.98***	.22
FESV experience of competency	−0.54***	.13
FESV mental distraction	−0.95***	.20
FESV countersteering activities	−0.49***	.01
FESV rest and relaxation	−1.95***	.43
SF-12 PC	−2.30***	.29
SF-12 MC	−2.37***	.31
VAS pain intensity	10.94***	.60
ODI	4.43***	.53
Days on sick leave	14.61***	.27

Range: FESV 4–24; helplessness and depression and anger 5–30; Range SF12, ODI, VAS: 0–100. Positive differences in VAS, ODI, days on sick leave and FESV helplessness and depression, anger and anxiety indicate an improvement. Negative differences in FESV action planning, cognitive restructuring, experience of competencies, mental distraction, countersteering activities, rest and relaxation and SF12 indicate an improvement

***p < .001 in dependent t-test

## Discussion

The present study indicates that patients with chronic back pain have lower utilization of health-care services, except for physiotherapy and psychotherapy, after a multidisciplinary rehabilitation in Germany than during the 6 months before, even if the effect sizes for the proven differences are at most small. After adjusting for various confounding variables, the changes in sick leave and psychological impairment after rehabilitation account for variance in the use of overall health-care services, general practitioners and specialists. Hence, the hypotheses are confirmed for subdimensions.

Patients in the present study had a lower use of general practitioners, specialists, massages and complementary therapists and were less often admitted to hospital after multidisciplinary rehabilitation in Germany. These results are in line with findings from different Scandinavian studies [18–20]. The utilization of physiotherapy and psychotherapy did not differ significantly before and after rehabilitation, although the means were reduced and the percentage of patients having at least one contact with a physiotherapist decreased from 67% to 49%. A possible explanation could be that active exercise therapy and psychotherapy are often recommended as follow-up care after rehabilitation [14]. The present results confirm that patients with chronic back pain in Germany have an increased use of health-care services, especially for physiotherapy, general practitioners, specialists and admission to hospital mainly before rehabilitation [6, 7, 35]. Massages are only recommended in combination with active exercise therapy for patients with chronic back pain [14] but were used by around 46% of the patients before rehabilitation. The decrease in massages seems to correspond with the implementation of guidelines, although around one-third of the patients still had massages after rehabilitation.

The effect sizes for the decrease in health-care utilization after multidisciplinary rehabilitation shown in the present study might be small. However, the results indicate important findings regarding direct costs due to chronic back pain that were also proved to be reduced after a special pain treatment [36]. The costs for a consultation with a general practitioner were estimated to be on average 60 euro in 2014 [37]. In the present study, this could be equalized by a reduction of 87.6 euro per patient only for the use of general practitioners compared to the 6 months before rehabilitation, which seems to be relevant for insurance and society.

In patients with chronic back pain, the results reveal a small influence of the changes due to rehabilitation on health-care utilization, particularly on the requirement for general practitioners and specialists. This influence is mainly explained by the reduction in days on sick leave and psychological impairment. Pain intensity, pain function and disability, and health-related quality of life also improved after rehabilitation (see Table 4). The changes in these variables and the medical characteristics themselves were not important to predict the utilization of health-care services.

The baseline utilization of health-care services had a great influence on the use after rehabilitation. Former health-care services use also seems to have influence on the effects of rehabilitation. Patients who had a higher use before rehabilitation gained less from a rehabilitation regarding general well-being, depression and pain intensity [35]. In the present study, a greater pain-related psychological impairment led to a higher utilization of health-care services after rehabilitation. A greater decrease in helplessness and depression involved fewer consultations with specialists, but in contrast a greater decrease in anxiety predicted a higher use of general practitioners and specialists. Cognitive coping capacities are improved at least for a short term after rehabilitation [38], but in the present study the effects for example in experience of competencies had no significant effect on health-care utilization.

In addition to the relevance of the decrease in days on sick leave, the results show the relevance of the number of days on sick leave for the use of all types of health-care services. Particularly for consultations with general practitioners, this could be explained through German regulations for sick leave. Compared to other European countries, Germany has a relatively high health-care utilization due to sick leave regulations [39], but the simultaneous reduction in utilization of health-care services and in days on sick leave after rehabilitation has also been shown in Scandinavian studies [18–20]. Additionally to the influence of days on sick leave and of the reduction in sick leave, patients who worked less overall used more health-care services and visited general practitioners more often. The relevance of sick leave and working status confirm findings that indicated differences in patients who stayed at work and those who were on sick leave regarding pain acceptance and mental health [17, 40]. The results are also in line with a study that showed a reduced sick leave after rehabilitation, while indicating differences between dysfunctional patients and adaptive copers in the long term [41].

Contrary to the hypothesis, age and socio-economic state had no influence on health-care utilization in the present study. These characteristics were relevant in the use of health-care services in chronic back pain patients [4], but they also had no effects on utilization of health-care services in hotel workers suffering from back pain [42].

Among psychological variables, activity-related fear-avoidance beliefs and fatalistic external control beliefs predicted health-care utilization after rehabilitation. Having fewer activity-related fear-avoidance beliefs led to a higher health-care utilization, which is in contrast to other researches [12, 42].

The reduction in health-care utilization, the influence of days on sick leave and the reduction in days of sick leave after rehabilitation on the use of health services indicate important findings regarding direct and indirect costs as well as the efficacy of rehabilitation [15]. But further research should also investigate whether other treatment options evoke similar effects. It has already been shown that a specific physiotherapeutic intervention provoked decreased health-care utilization. However, the decrease was smaller than after multidisciplinary rehabilitation [20].

It is important to point out, that due to characteristics of financing and regulation for rehabilitation, but also to others such as sick leave, the results of this study depend on the German health-care system and may not be transferable to other health-care systems. Some further limitations need to be mentioned. Due to regression analyses only correlations were investigated; cause and effects cannot be specified. Furthermore, the assumption of homoscedasticity was not met for the outcome variables in the regression analyses, hence, any generalization of the results has to be made with caution. A strength of the study is the large sample size but the dropout rate 6 months after rehabilitation was high (30.9%). With regard to the differences between study patients and dropout patients, described in the methods section, and in addition to the rate of decliners (32.9%), the results cannot be supposed to be representative for all patients with chronic back pain after a multidisciplinary rehabilitation in Germany.

The study was based on a secondary analysis of an existing data set, therefore some variables could not be considered: there is only information on how often patients have consultations, but no reasons for the utilization of the respective health-care service are reported. The study does not examine different types of specialists or insurance that are reported to be relevant for care-seeking behaviour [6]. Depression, anxiety and stress have an influence on quality of life and health-care utilization in patients with chronic back pain [12]. Depression and anxiety are only measured with FESV. For further research a more detailed assessment of depression, anxiety and stress in patients with chronic back pain and their influence on the use of health-care services could be of interest. This also applies to a more detailed assessment of co-morbidities both before rehabilitation and 6 months after rehabilitation.

In this study only questionnaires based on patients’ or physicians’ disclosure were used. This could lead to a common method bias [43]. However, the bias should be controlled by the sequence of the questions and the anonymity. In addition, this kind of bias only has influence in the case of severe violation. It should be noticed that cognitive processes such as recall bias seem to affect patients with chronic back pain and many fear-avoidance beliefs in terms of their experience of pain, which could also lead to biased results. However, regarding this bias, there is no difference between patients with chronic back pain and healthy controls [44].

What does the present paper contribute to the current literature? To the best of our knowledge, there is no other study concerning the situation in Germany that has investigated the effect of multidisciplinary rehabilitation in relation to health care utilization in such a large cohort of patients suffering from back pain. The papers discussed above [18–20] involve Scandinavian countries and The Netherlands, and rely on lower sample sizes. Unlike the rehabilitation systems in those countries, the German system is characterized by a high extent of standardization and a rehabilitation intervention lasting 3 weeks. Outpatient rehabilitation is a day-long, ambulant intervention that differs only slightly in its content and duration from an inpatient intervention.

Our study’s findings therefore reveal that the perceptible but minor effects on health care utilization appear to be independent of the rehabilitation system, and that they also apply to Germany’s rather rigid system whereby rehabilitation must usually be implemented on a short-term and intensive basis.

Furthermore, unlike the studies cited above, in this research we investigated the factors affecting health care utilization. The great importance we demonstrated of psychological variables such as pain-related helplessness, depression and the externality of control beliefs could help explain why the treatment-effect sizes on health care utilization were different in the aforementioned studies. Their patient cohorts may have exhibited different distribution characteristics regarding those variables.

## Conclusions

The use of health-care services, except of physiotherapy and psychotherapy, in patients with chronic back pain is significantly lower 6 months after rehabilitation in Germany than before rehabilitation. After controlling various variables, the overall health-care utilization and the use of physicians could be explained by the reduction in sick leave and in psychological impairment after rehabilitation. The baseline values of health-care services use, days on sick leave, state of health and psychological impairment were the most important predictors of health-care use in patients with chronic back pain after rehabilitation. The study confirms findings of reduced health-care utilization after rehabilitation having an important impact on costs due to back pain.
